# Examining the performance of the International Trauma Questionnaire with clinical checks in a multinational European sample

**DOI:** 10.1371/journal.pone.0360940

**Published:** 2026-10-21

**Authors:** Philip Hyland, Mark Shevlin, Menachem Ben-Ezra, Marylène Cloitre, Matus Adamkovic, Agata Błachnio, Marek Preiss, Ella Salgo, Mihaela Fadgyas Stanculete, Zsolt Szabolcs Unoka, Thanos Karatzias

**Affiliations:** 1 Department of Psychology, Maynooth University, Kildare, Ireland; 2 School of Psychology, Ulster University, Derry, Northern Ireland; 3 School of Social Work, Ariel University, Ariel, Israel; 4 Silver School of Social Work, New York University, New York, New York, United States of America; 5 Institute of Social Sciences CSPS, Slovak Academy of Sciences, Košice, Slovakia; 6 Faculty of Education, Charles University, Prague, Czechia; 7 Faculty of Humanities and Social Sciences, University of Jyväskylä, Jyväskylä, Finland; 8 John Paul II Catholic University of Lublin, Lublin, Poland; 9 National Institute of Mental Health, Klecany, Czech Republic; 10 Department of Psychiatry and Psychotherapy, Semmelweis University, Budapest, Hungary; 11 Department of Neurosciences, Discipline of Psychiatry and Pediatric Psychiatry, Iuliu Hatieganu University of Medicine and Pharmacy, Cluj-Napoca, Romania; 12 Department of Psychiatry and Psychotherapy, Semmelweis University, Budapest, Hungary; 13 Edinburgh Napier University, School of Health and Social Care, Edinburgh, Scotland; Hochschule Niederrhein - Campus Mönchengladbach: Hochschule Niederrhein - Campus Monchengladbach, GERMANY

## Abstract

**Background:**

The International Trauma Questionnaire (ITQ) is a widely used self-report measure of PTSD and Complex PTSD (CPTSD). ‘Clinical checks’ – brief follow up questions verifying respondent comprehension of the symptom item – have recently been developed for the ITQ to reduce false-positive diagnostic cases. This study tested whether the ITQ with clinical checks generates psychometrically sound scores and adds clinically relevant information to standard symptom assessment.

**Methods:**

Data (*N* = 5,070) were collected using quota-based sampling procedures from adults living in five European countries (Czech Republic, Poland, Hungary, Romania, and Slovakia).

**Results:**

Overall, 82.2% of respondents were trauma-exposed, and 19.2% met diagnostic requirements for PTSD (7.2%) or CPTSD (12.0%) on the standard ITQ. With clinical checks, these fell to 9.8% overall, 3.9% for PTSD, and 5.9% for CPTSD. The ITQ with clinical checks produced scores with excellent factorial validity, internal reliability, and latent classes corresponding to PTSD and CPTSD. Associations with clinically relevant variables including suicidality and anxiety-depression symptoms were stronger with than without the clinical checks. Females, those with more extensive trauma histories, and higher concurrent anxiety-depression symptoms were more likely to retain than lose their diagnostic status when the clinical checks were used.

**Conclusions:**

The ITQ with clinical checks generated scores with excellent psychometric properties, captured additional clinically relevant information, and yielded more plausible prevalence estimates compared to the standard ITQ scores. The potential value and limitations of using clinical checks in self-report questionnaires are discussed.

## Introduction

The International Trauma Questionnaire (ITQ) [1] is a self-report measure of Posttraumatic Stress Disorder (PTSD) and Complex PTSD (CPTSD) as these disorders are defined in the 11^th^ revision of the *International Classification of Diseases* (ICD-11) [2]. The scale includes 18 items: 12 items measuring symptoms from across six clusters – three reflecting PTSD (re-experiencing in the here and now, avoidance, sense of current threat) and three reflecting ‘disturbances in self-organisation’ (DSO: affective dysregulation, negative self-concept, and disturbed relationships) – and six items measuring functional impairments related to both symptom sets. The ITQ can be used to generate symptom severity scores and to identify individuals who meet diagnostic requirements for *ICD-11* PTSD and CPTSD. To date, the scale has been translated into 38 languages, and the original validation study has been cited over 1,800 times, demonstrating its value to researchers and clinicians worldwide.

The ITQ underwent extensive psychometric testing during its development [3] and following the release of the finalised version [4,5]. Dozens of studies using mixture modelling techniques showed that the ITQ identifies distinct groups of people with symptom profiles reflective of PTSD and CPTSD, while similar numbers of studies using confirmatory factor analysis demonstrated that the latent structure of the scale items aligned with the theoretical distinction between the PTSD and DSO symptoms (see [4] for a review). With considerable evidence supporting the psychometric properties of the ITQ scores, the scale began to be used in epidemiological studies to estimate the prevalence rates of *ICD-11* PTSD and CPTSD in the general adult population. Table 1 lists prevalence rates obtained from 12 studies that used either probability-based sampling or quota-matched sampling to construct representative samples of the general adult population. Estimates varied considerably across countries and were lower in studies using probability-based sampling. Across all studies, 5.0% met requirements for PTSD and a further 5.0% met requirements for CPTSD.

**Table 1:** Summary of prevalence rates of ICD-11 PTSD and CPTSD using the ITQ in nationally representative samples.

Country	Lead author (Year)	Sampling method	PTSD	CPTSD	Total
Germany*	Maercker et al. [6]	Probability-based	1.5%	0.5%	2.0%
USA	Cloitre et al. [7]	Probability-based	3.4%	3.8%	7.2%
Israel	Hyland et al. [8]	Quota-based	6.7%	4.9%	11.2%
Ireland	Hyland et al. [9]	Quota-based	5.0%	7.7%	12.7%
Germany	Kampling et al. [10]	Probability	1.4%	2.5%	3.9%
Lithuania	Kvedaraite et al. [11]	Quota-based	5.8%	1.8%	7.6%
Ukraine	Martsenkovskyi et al. [12]	Quota-based	11.1%	9.2%	20.3%
Hong Kong	Ho et al. [13]	Probability-based	1.7%	4.2%	5.9%
Ireland	McGinty et al. [14]	Quota-based	2.4%	8.8%	11.2%
Ukraine	Wang et al. [15]	Quota-based	14.4%	8.9%	23.3%
Denmark	Jørgensen et al. [16]	Probability-based	4.7%	1.9%	6.6%
Netherlands	Hoeboer et al. [17]	Probability-based	1.0%	1.6%	2.6%
United Kingdom	Shevlin et al. [18]	Quota-based	5.4%	9.5%	14.9%
**Mean**			**5.0%**	**5.0%**	**10.0%**

*Note*. * = based on a very narrow definition of trauma exposure.

Prevalence estimates derived from self-report questionnaires are assumed to overestimate true prevalence rates [19]. The main evidence for this is that prevalence rates (of PTSD and other psychiatric disorders) derived from self-report questionnaires tend to be higher than estimates derived from clinician-administered diagnostic interviews [20–25]. Self-report questionnaires are typically designed to maximise diagnostic sensitivity (i.e., of those who have the disorder, correctly identifying as many as possible as diagnostic cases), meaning that some number of false-positive diagnostic cases are inevitable. Some false-positive symptom endorsements are likely to be caused by respondents not fully understanding the intended meaning of a symptom item and mistakenly reporting normal distress as a symptom of psychopathology. Clinician-administered interviews are designed to offset this possibility by allowing the interviewer to clarify misunderstandings and gather more information to better discriminate symptoms of psychopathology from normal distress. In this way, clinical interviews are typically designed to maximise diagnostic specificity (i.e., of those who do not have the disorder, correctly identifying as many as possible as non-diagnostic cases) and, in turn, positive predictive value (i.e., of those identified as diagnostic cases, the proportion who actually do have the disorder).

Ideally, self-report questionnaires and clinical interviews are used in combination to identify diagnostic cases [19]. However, in many clinical and research settings this is not possible due to time, financial, and/or human-resource constraints. As such, many researchers and clinicians rely solely on self-report questionnaires, accepting that obtained prevalence rates are likely to be somewhat overestimated. Recognizing these resource constraints, as well as the many inherent advantages of using self-report questionnaires over clinical interviews to assess psychological phenomena [26], Shevlin et al. [27] developed a novel assessment method that incorporates the best elements of the self-report and clinical interview methods. This approach involves modifying a standard self-report questionnaire (e.g., the ITQ) so that every symptom item includes a follow-up question that *checks* whether the respondent correctly understood the intended *clinical meaning* of the item and responded accordingly. An example is as follows: the ITQ assesses for an exaggerated startle response by asking respondents how bothered they have been in the past month by ‘*Feeling jumpy or easily startled?*’ The associated clinical check states: ‘*Something normal, like a noise, can shock and set your heart racing* – *something that doesn’t bother other people. Does this happen to you?’* The clinical checks are intended to serve a similar function to the additional questions that interviewers can use to supplement an initial symptom assessment. They are intended to confirm symptom presence and thereby screen out false-positive symptom endorsements. Ultimately, they are intended to remove false-positive diagnostic cases and generate estimates of prevalence that more closely approximate the true prevalence rate.

Shevlin et al. [27] first demonstrated the utility of using clinical checks in a proof-of-principle study focused on the assessment of prolonged grief disorder. Subsequently, clinical checks were developed for the ITQ by an international team of researchers and clinicians, including the ITQ developers. Shevlin et al. [18] trialled their use in a quota-matched representative sample of adults living in the United Kingdom (UK: *N* = 975). The proportion of people meeting diagnostic requirements for *ICD-11* PTSD or CPTSD dropped from 14.9% to 8.7% when the clinical checks were used (relative reduction = 41.6%). The rate of PTSD dropped from 5.4% to 3.8% (relative reduction = 29.6%) while the rate of CPTSD dropped from 9.5% to 4.9% (relative reduction = 48.4%). The authors described these reductions as falling within the ‘Goldilocks zone’; not too small to wonder if the addition of the clinical checks was worth the extra time and participant burden, and not too large to wonder if people with genuine clinical distress were being systematically excluded. They argued that the clinical checks resulted in more plausible estimates of the true prevalence rates of *ICD-11* PTSD and CPTSD in the general adult population of the UK.

These initial findings from Shevlin et al. [18] suggest that adding clinical checks to the ITQ may be of value to researchers and clinicians whose primary interest is in identifying probable diagnostic cases, but much remains to be investigated before these clinical checks can be used with confidence. This study was designed to rigorously test various assumptions about the nature, value, and validity of using the ITQ with clinical checks. Using data from a large, multinational European sample (*N* = 5,070), five research objectives were developed.

The first was to test the psychometric properties of the scores obtained using the ITQ with clinical checks. Since the clinical checks are intended to measure clinically relevant information beyond that which is measured by the standard ITQ items, it cannot simply be assumed that these scores will possess the same strong psychometric properties as the standard ITQ scale scores. This must be evidenced. We therefore tested whether (a) the latent structure of the ITQ with clinical checks corresponds to the *ICD-11* description of PTSD and CPTSD, (b) the resulting scale scores possess satisfactory estimates of internally reliability, and (c) distinct groups of people with response patterns reflecting the distinction between PTSD and CPTSD are identifiable.

The second objective was to examine what effect the clinical checks have on endorsement rates at the level of symptoms, symptom clusters, and disorders (i.e., *ICD-11* PTSD and CPTSD). Because the clinical checks can only reclassify a symptom from present to absent, some reduction is guaranteed; the question of interest is whether the magnitude of that reduction is large enough to justify the additional participant burden, yet not so large as to suggest that participants with significant distress are being systematically screened out.

The third objective was to test the assumption that the clinical checks add clinically relevant information to the assessment of PTSD and DSO symptoms. This may not be the case. The clinical checks might just represent a complicated way of increasing the intensity threshold needed for symptom endorsement. This was tested by comparing the proportion of people meeting diagnostic requirements for *ICD-11* PTSD or CPTSD under the clinical checks with the proportion meeting requirements when the threshold for symptom endorsement on the standard ITQ was raised by one Likert-scale point (i.e., moving from 2 to 3 on the Likert scale). If these proportions are similar, and the same people are identified, this would indicate that the clinical checks offer nothing beyond what a simple threshold adjustment achieves.

The fourth objective was to test whether diagnostic classifications derived using the clinical checks are more strongly associated with clinically relevant external criterion variables than classifications derived without them. If the clinical checks increase the probability of identifying true positive cases of *ICD-11* PTSD and CPTSD, then diagnostic status estimated with the clinical checks should show stronger associations with lifetime history of suicidal ideation, history of attempted suicide, and anxiety-depression symptoms.

The fifth and final objective was exploratory. If the clinical checks function as intended, there will be a group of people who screen positive for *ICD-11* PTSD or CPTSD on the standard ITQ but do not do so once the clinical checks are applied. We examined what demographic, trauma-related, and psychological variables were associated with losing versus retaining probable diagnostic status under the clinical checks.

## Materials and methods

### Participants and procedures

Data, materials, code, and online resources used for this study are available at https://osf.io/37eac/overview. Based on an assumed, combined prevalence rate of ~10% for PTSD and CPTSD, an *a priori* sample size calculation was conducted to determine the minimum sample size needed for a margin of error of ±1% and a 95% confidence level. This resulted in a required sample size of 3,342. Participants in this study (*N* = 5,070) were adults living in five central and eastern European countries: Czech Republic (*n* = 1,017), Hungary (*n* = 1,009), Poland (*n* = 1,022), Romania (*n* = 1,007), and Slovakia (*n* = 1,015). Participants were recruited between July 7 and 28, 2025, by *TGM Research*. Identical procedures were used to recruit participants in each nation. Eligible participants were adults aged 18–65 years residing in the relevant country and capable of completing the survey in their national language. Participants were recruited from opt-in panels of survey participants using quota sampling procedures based on national level sex, age, and geographical distributions. Given the non-probability nature of participant selection, we do not claim these samples are fully representative of the populations from which they were drawn, but merely that steps were taken to construct samples that are reasonably representative of the different adult populations across these demographic variables.

Completion rates among those who initiated the survey ranging from 35.4% to 59.2%. All surveys included multiple attention checks, and *TGM Research* uses several methods to prevent and screen out invalid responses (e.g., from bots, duplicate IP addresses, suspicious patterns of responding, and unrealistically fast completion times). Sample participants passed all quality control checks. Ethical approval for the collection of the data was first obtained from the research ethics committee at Ariel University in Israel (home institution of the lead investigator of the project [MBE]). Subsequently, members of the research team based in each nation obtained ethical approval from their own home institution. Written informed consent was obtained from all participants. Sociodemographic information for the participants is presented in Table 2 (racial/ethnic information were not collected).

**Table 2:** Demographic Information on the Sample Participants (N = 5070).

	% (n)	Mean (SD)
*Nation*		
Czech Republic	20.1 (1017)	--
Hungary	19.9 (1009)	--
Poland	20.2 (1022)	--
Romania	19.9 (1007)	--
Slovakia	20.0 (1015)	--
Age	--	41.29 (12.69)
*Sex*		
Males	49.7 (2518)	--
Females	50.3 (2552)	--
*Marital Status*		
Single	24.9 (1264)	--
In relationship (not cohabiting)	5.5 (277)	--
In relationship (cohabiting)	21.5 (1088)	--
Married	38.1 (1933)	--
Separated but legally married	0.7 (33)	--
Divorced	7.6 (384)	--
Widowed	1.8 (91)	--
Number of children	--	1.10 (1.13)
*Highest Education Attainment*		
Completed mandatory schooling	4.5 (228)	--
Completed general/secondary school	28.0 (1419)	--
Completed vocational school	35.1 (1779)	--
Completed university	32.4 (1644)	--
*Employment Status*		
Employed	77.6 (3936)	--
Unemployed	9.7 (494)	--
Student	4.5 (226)	--
Retired	5.4 (276)	--
Not working due to disability	2.7 (138)	--
*Religious Affiliation*		
No religion (Atheist/Agnostic)	28.7 (1453)	--
Christian	65.2 (3308)	--
Hindu	0.2 (10)	--
Sikh	0.1 (4)	--
Jewish	0.2 (10)	--
Muslim	0.3 (14)	--
Buddhist	0.5 (26)	--
Other	4.8 (245)	--
*Annual Income (EURO)*		
€0-€9,999	25.0 (1265)	--
€10,000-€19,999	32.3 (1638)	--
€20,000-€29,999	17.3 (875)	--
€30,000-€39,999	7.8 (393)	--
€40,000-€49,999	5.4 (273)	--
€50,000-€59,999	3.3 (166)	--
€60,000-€69,999	2.2 (113)	--
€70,000-€79,999	2.1 (109)	--
€80,000-€89,999	1.7 (85)	--
€90,000 or more	3.0 (153)	--
*Financial Worries*		
Not worried at all	9.8 (497)	--
A little worried	32.7 (1658)	--
Moderately worried	31.4 (1594)	--
Very worried	17.9 (905)	--
Extremely worried	8.2 (416)	--
Life satisfaction	--	59.64 (26.00)
Anxiety-depression	--	4.03 (3.25)

### Measures

*Translation information.* Survey materials not already available in the respective languages were translated from English according to WHO [28] guidelines. This involved a forward-translation, review, and back-translation by different individuals, followed by a review of the materials by team members who were involved in the development of the different questionnaires. A final review was conducted by research team members before the soft launch of the survey (i.e., collection of data from 25–50 people in each nation to ensure that the survey materials were understandable and could be completed by the participants without difficulties). No changes were made between the soft launch and the full launch.

#### Primary measures.

*Trauma exposure*: The International Trauma Exposure Measure (ITEM) [29] assesses trauma exposure in accordance with the *ICD-11* definition (i.e., any event provoking feelings of extreme threat or horror). The ITEM assesses exposure to 21 potentially traumatic life events during childhood (0–12 years), adolescence (13–18 years), and adulthood (19 years and older). Respondents were asked if to indicate if they had experienced each event during each developmental. An additional item asked respondents to describe any other potentially traumatic event not listed. Respondents were then asked to nominate the event that caused them the most distress (i.e., their index trauma) and how long ago this event occurred (< 1 month ago, 1–6 months ago, 6–12 months ago, 1–5 years ago, 6–10 years ago, and > 10 years ago). The ITEM is available at https://www.traumameasuresglobal.com/item.

*ICD-11 PTSD and CPTSD*: All participants who experienced at least one traumatic life event completed the ITQ with clinical checks (ITQ-CC) [18], anchored to their nominated index trauma event. Participants rated how bothered they have been by each PTSD symptom over the past month, and how true each DSO symptom is of them, on a five-point Likert scale (0 = *Not at all*, 1 = *A little bit*, 2 = *Moderately*, 3 = *Quite a bit*, 4 = *Extremely*). By convention for all self-report and interview-based assessments for PTSD/CPTSD, a rating of 2 or higher was used to indicate symptom endorsement. Participants who rated a symptom 2 or higher were then presented with the associated clinical check and answered these using a Yes-No format. All 12 symptom items had an associated clinical check, and one clinical check was used for each set of functional impairment items. Under the ITQ-CC scoring rules, a symptom was present based on a symptom rating of 2 or higher followed by endorsement of the corresponding clinical check. The ITQ-CC is available at https://www.traumameasuresglobal.com/ptsd.

Diagnostic requirements for PTSD were met if the index trauma occurred more than one month ago, at least one symptom was present in the re-experiencing, avoidance, and sense of current threat clusters, and there was evidence of functional impairment related to these symptoms. Diagnostic requirements for CPTSD were met if all PTSD requirements were satisfied plus at least one symptom was present from the affective dysregulation, negative self-concept, and disturbed relationship symptom clusters, and there was evidence of functional impairment related to these symptoms.

#### Secondary measures.

*Suicidality*: Lifetime history of suicidal ideation and lifetime history of attempted suicide were assessed using two items from the 2014 Adult Psychiatric Morbidity Survey [30]. Suicidal ideation was assessed with the item: ‘*There may be times in everyone’s life when they become very miserable and may feel like taking drastic action because of these feelings. Have you ever thought of taking your life, even if you would not really do it?*’ Attempted suicide was assessed with the item: ‘*Have you ever made an attempt to take your own life?*’ Both were answered using a Yes–No format. These items were not administered in the Czech Republic survey due to an ethical restriction, and analyses involving suicidality are therefore based on participants from the remaining four countries (*n* = 4,053).

Anxiety-depression symptoms: The four-item Patient Health Questionnaire scale (PHQ-4) [31] is a measure of core symptoms of generalized anxiety (persistent worry and nervousness) and depression (persistent low mood and apathy). Participants rated how often they have been bothered by these symptoms over the last two weeks on a four-point Likert scale (0 = *Not at all*, 1 = *Several days*, 2 = *More than half the days*, 3 = *Nearly every day*). Scores can range from 0−12 with higher scores indicating higher levels of anxiety-depression. According to the scale developers, scores from 3−5 indicate ‘mild’ symptoms, 6−8 ‘moderate’ symptoms, and 9−12 ‘severe’ symptoms. A recent systematic review of 26 studies from 19 countries found strong support for the reliability and validity of the PHQ-4 scores [32]. The internal reliability of the scale scores in this sample was excellent (α = .88).

### Analytic plan

The analytic plan involved several steps to address each objective, and all analyses were conducted in Mplus version 8.2 [33] and SPSS v29.

*Objective 1: Psychometric properties of the ITQ-CC.* Confirmatory factor analysis (CFA) was used to test if the latent structure of the ITQ-CC items was consistent with the *ICD-11* description of PTSD and CPTSD. Three models were tested based on the existing and extensive literature for the standard ITQ [4,5]. Model 1 was a correlated six-factor model (re-experiencing in the here and now, avoidance, sense of current threat, affective dysregulation, negative self-concept, disturbed relationships), with two items loading onto each factor. Model 2 was a correlated two-factor second-order model in which a higher-order PTSD factor explained the covariation between the first three first-order factors and a higher-order DSO factor explained the covariation between the remaining three first-order factors. Model 3 was a modified version of model 2 where the first-order affective dysregulation factor was allowed to cross-load on the second-order PTSD factor. This model was based on several findings showing that the affective dysregulation factor can load onto the higher-order DSO factor above a value 1.0 [34,35], because of unmodelled variance in the first-order affective dysregulation factor due to the second-order PTSD factor [36].

Due the binary nature of the ITQ-CC items, these models were estimated using the weighted least squares mean- and variance-adjusted estimator [37]. Model fit was assessed according to guidelines and recommendations for tests of exact and approximate fit [38,39]. Exact model fit was assessed using the chi-square test (*χ*^*2*^) and supported by a non-significant result, but it is well established that good fitting models can be rejected when sample sizes are large [40]. Approximate fit was tested using the comparative fit index (CFI), Tucker-Lewis index (TLI), root-mean square error of approximation (RMSEA), and standardized root-mean square residual (SRMR). The CFI and TLI range from 0–1 where values closer to 1 indicate better fit (and values greater than.90 and.95 are generally regarded as indicating ‘acceptable’ and ‘excellent’ fit, respectively). The RMSEA and SRMR range from 0 to infinity where values closer to 0 indicate better fit (and values less than.08 and.06 are generally regarded as indicating ‘acceptable’ and ‘excellent’ fit, respectively). Direct model comparisons were tested using a *χ*^*2*^ difference test using the DIFFTEST function in Mplus. Upon determining the optimal latent structure of the ITQ-CC scale items, the internal reliability of the scale scores was estimated using omega reliability (ω) where values can range from 0–1 and higher values indicate higher internal reliability.

Latent class analysis (LCA) was performed to determine the optimal number of classes needed to explain the ITQ-CC data, and to determine if distinct groups of people with symptom profiles reflective of *ICD-11* PTSD and CPTSD were identifiable. Consistent with most LCA studies using the ITQ [4], the observed variables were six binary indicators representing whether participants met requirements for each symptom cluster. Models with one to six classes were estimated using robust maximum likelihood estimation, using 500 random start values followed by 100 final stage optimizations to avoid solutions based on local maxima. Relative fit was compared using the Akaike Information Criterion (AIC), the Bayesian Information Criterion (BIC), and sample size adjusted Bayesian Information Criterion (ssaBIC), where the model with the lowest value is preferred. Simulation studies indicate that the BIC and ssaBIC should be prioritised in selecting the optimal class solution [41]. Additionally, the Lo-Mendell-Rubin adjusted likelihood ratio test (LMR-A) was used to determine the appropriate number of classes. A non-significant result indicates that the model with one less class should be favoured. For the best fitting model, entropy and average posterior probability values were used to describe how well the classes were differentiated and how well participants were assigned to their class, respectively. Both values can range from 0–1, and values above.70 are generally regarded as satisfactory [41].

*Objectives 2 and 3: Effect of clinical checks on endorsement rates:* The proportions meeting requirements for each symptom, symptom cluster, and disorder with and without the clinical checks were compared using McNemar’s *Z*-test. This test was also used to compare proportions meeting diagnostic requirements for PTSD, CPTSD, and either disorder based on the ITQ-CC scoring method (i.e., a rating of ≥ 2 on the Likert scale plus endorsement of the clinical check) and on the standard ITQ when the threshold for symptom endorsement was raised from 2 (‘Moderately’) to 3 (‘Quite a bit’) on the Likert scale. Cohen’s kappa (κ) values were calculated to determine agreement in the specific individuals identified by each scoring method. κ values range from 0−1 and are commonly interpreted as slight (.00−.20), fair (.21−.40), moderate (.41−.60), substantial (.61−.80), and almost perfect (.81–1.00) agreement [42].

*Objective 4: Associations with external criterion variables.* Pearson *χ*^*2*^ tests were used to examine the strength of the associations between variables representing *ICD-11* PTSD and CPTSD diagnostic status with and without the clinical checks and lifetime history of suicidal ideation, lifetime history of attempted suicide, and scoring within the ‘severe’ range on the PHQ-4. Odds ratios (OR) were used to quantify the magnitude of these associations.

*Objective 5: Correlates of losing versus retaining probable diagnostic status*. Finally, binary logistic regression was used to identify variables associated with losing diagnostic status for *ICD-11* PTSD or CPTSD when the clinical checks were used. The criterion variable had two levels (0 = those whose probable diagnostic status was unchanged when the clinical checks were used, and 1 = those who no longer met diagnostic requirements when the clinical checks were used). Predictor variables were sex (0 = male, 1 = female), age, marital status (0 = currently in a committed relationship, 1 = not currently in a committed relationship), number of children, education status (0 = no university education, 1 = university education), employment status (0 = currently employed, 1 = currently unemployed), annual income, time since index trauma, number of different trauma types experienced, anxiety-depression symptoms, and country of residence (Czech Republic was treated as the reference category).

## Results

### Descriptive statistics

Among all participants, 82.2% (*n* = 4,170) experienced at least one traumatic event in their lifetime, and the mean number of different events experienced was 4.97 (*Mdn* = 4.00, SD = 4.80). Among exposed participants, the most commonly reported index trauma event was ‘someone close to you was diagnosed with a life-threatening illness or experienced a life-threatening accident’ (20.8%, *n* = 867), and the most common timeframe was ‘more than 10 years ago’ (46.6%, *n* = 1944).

Of those administered the suicidality items, 31.0% (*n* = 1,256) reported a lifetime history of suicidal ideation, and 11.4% (*n* = 461) reported a lifetime history of an attempted suicide. The mean PHQ-4 score was 4.03 (*Mdn* = 4.00, SD = 3.25), and 11.0% (*n* = 558) scored in the ‘severe’ range, indicative of clinically significant levels of anxiety-depression symptoms.

### Objective 1 results

The CFA model fit results are presented in Table 3. Model 1, the correlated six-factor model, converged normally and fit the sample data closely. Model 2, the two-factor second-order model, also fit the sample data closely but the affective dysregulation factor loaded onto the DSO factor at a value of 1.02 (i.e., an out-of-bounds parameter estimate). Model 3, which allowed the affective dysregulation factor to load onto both second-order factors, converged normally with no out-of-bounds parameter estimates and was a close fit to the sample data. Nevertheless, Model 1 was a closer fit to the sample data than Model 3 (DIFFTEST scaled Δ*χ*^*2*^ = 62.85, df = 7, *p* < .001) and was therefore favoured as the optimal representation of the latent structure of the ITQ-CC items in this sample.

**Table 3:** Confirmatory factor analysis results for the International Trauma Questionnaire with clinical checks (N = 5070).

	*χ* ^*2*^	df	p	CFI	TLI	RMSEA (90%CI)	SRMR
Model 1. Six-factor model	103.720	39	<.001	.999	.998	.018 (.014,.022)	.014
Model 2. Second-order model	332.841	47	<.001	.995	.993	.035 (.031,.038)	.026
Model 3. Modified second-order model	185.977	46	<.001	.997	.996	.024 (.021,.028)	.020

*Note*. *χ*^*2*^ = chi-square test; df = degrees of freedom; p = statistical significance; CFI = Comparative Fit Index; TLI = Tucker-Lewis Index; RMSEA (90% CI) = Root Mean Square Error of Approximation with 90% confidence intervals; SRMR = Standardized Root-Mean Square Residual; Estimator = WLSMV.

The factor loadings and factor correlations for Model 1 are presented in Table 4. All loadings were positive and strong, ranging from.78 to.96 (all *p*s < .001). All factors were positively and strongly correlated with one another, ranging from.61 to.93 (all *p*s < .001). Internal reliability was satisfactory for each set of items: re-experiencing (ω = .86), avoidance (ω = .90), sense of current threat (ω = .87), affective dysregulation (ω = .78), negative self-concept (ω = .95), and disturbed relationships (ω = .90).

**Table 4:** Standardized factor loadings and factor correlations (N = 5070).

	RE	AV	TH	AD	NSC	DR
***Factor Loadings***						
Upsetting dreams	.82					
Reliving in the here and now	.92					
Avoiding internal reminders		.90				
Avoidance external reminders		.91				
Being alert, on guard, watchful			.88			
Feeling jumpy or easily startled			.88			
Long time to calm down when upset				.78		
Feel numb or emotionally shut down				.82		
Feel like a failure					.95	
Feel worthless					.96	
Feel cut-off from others						.91
Difficulty staying close to others						.91
**Factor correlations**						
Re-experiencing in the here and now (RE)	1					
Avoidance (AV)	.77	1				
Sense of current threat (TH)	.82	.80	1			
Affective dysregulation (AD)	.79	.76	.93	1		
Negative self-concept (NSC)	.67	.61	.79	.88	1	
Disturbed relationships (DR)	.66	.68	.78	.92	.89	1

*Note*. All factor loadings and correlations are statistically significant (*p* < .001).

The LCA results are shown in Table 5. The BIC and ssaBIC values were lowest for the four-class solution, and the LMR-A test became non-significant when five classes were extracted, thereby supporting a four-class solution. The entropy value for the four-class model was.73, and average posterior probabilities were.91 (class 1),.85 (class 2),.75 (class 3), and.75 (class 4), indicating acceptable classification precision (Fig 1).

**Table 5:** Latent class analysis model fit statistics (N = 5070).

Classes	Log likelihood	AIC	BIC	ssaBIC	LMR-A (*p*)	Entropy
1	−18756.288	37524.577	37563.763	37544.697	--	--
2	−14880.576	29787.152	29872.056	29830.746	7623.762 (<.001)	.84
3	−14611.586	29263.172	29393.794	29330.241	529.119 (<.001)	.75
**4**	**−14492.370**	**29038.739**	**29215.079**	**29129.282**	**234.505 (<.001)**	**.73**
5	−14479.348	29026.697	29248.754	29140.713	25.614 (.079)	.77
6	−14470.413	29022.826	29290.601	29160.316	17.577 (.087)	.73

*Note*. AIC: Akaike Information Criterion, BIC: Bayesian Information Criterion (BIC), ssaBIC: sample-size adjusted BIC, (LMR-A): Lo-Mendell-Rubin adjusted likelihood ratio test; Best fitting model in bold.

**Fig 1:**
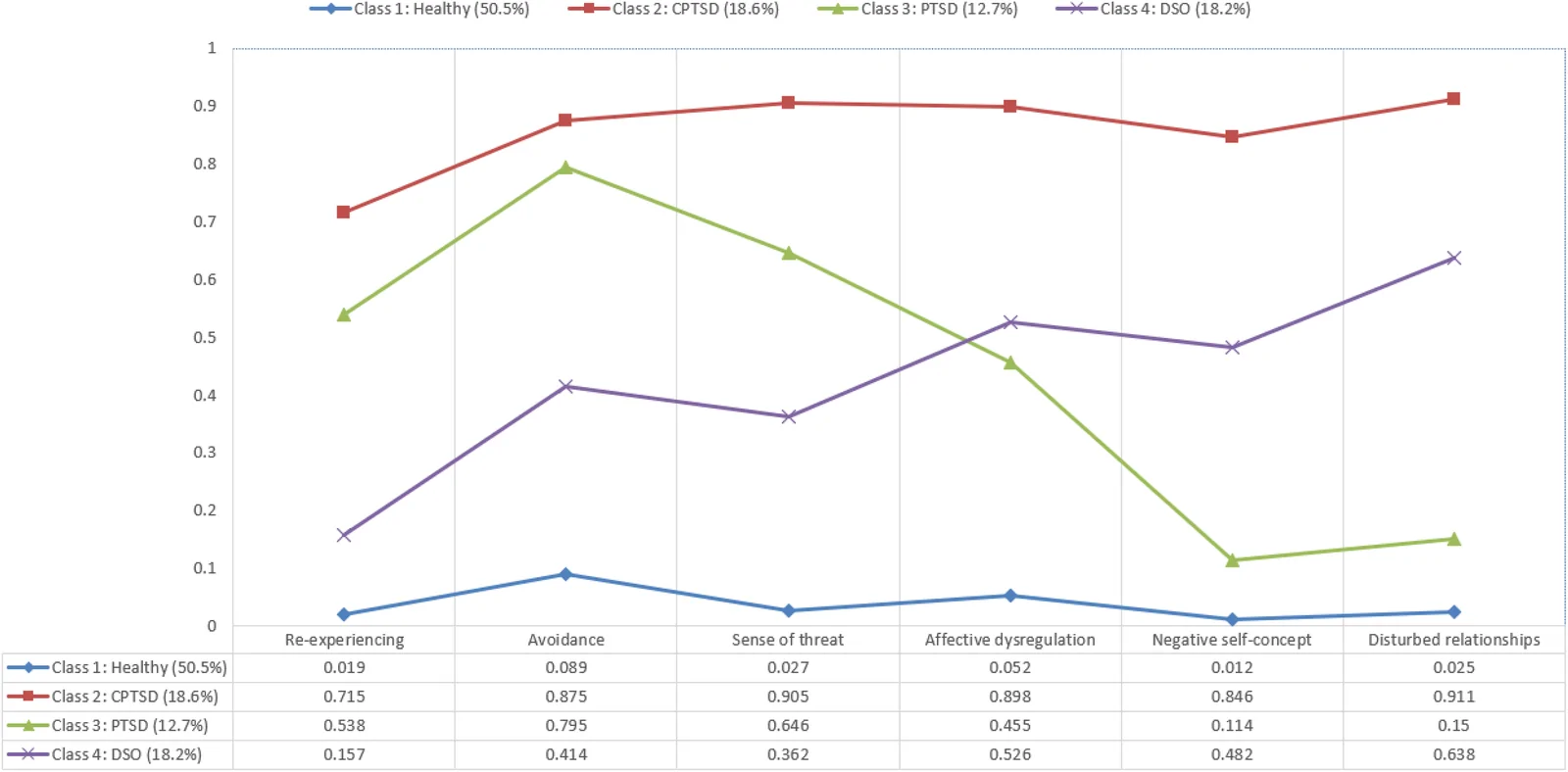
The four classes.

Class 1 (50.5%) was characterised by low probabilities across all six symptom clusters and was labelled the ‘healthy class’. Class 2 (18.6%) was characterised by high probabilities of endorsing all symptom clusters and was labelled the ‘CPTSD class’. Class 3 (12.7%) was characterised by moderate-to-high probabilities of endorsing all PTSD symptom clusters, a low-to-moderate probability of endorsing the affective dysregulation symptom cluster, and very low probabilities of endorsing the other DSO symptom clusters; this was labelled the ‘PTSD class’. Finally, Class 4 (18.2%) was characterised by low probabilities of endorsing the PTSD clusters and moderate probabilities of endorsing the DSO clusters and was labelled the ‘DSO class’.

### Objective 2 results

The proportions endorsing each symptom, symptom cluster, and disorder requirements with and without the clinical checks are presented in Table 6.

**Table 6:** Symptom, symptom cluster, and disorder prevalence rates with and without clinical checks (CC) (N = 5070).

	Endorsement		Endorsement + CC		Decreases in cases		
	N	%	N	%	N	%	McNemar’s Z (p)
***Symptoms***							
Nightmares	1335	26.3	646	12.7	689	51.6	26.25 (<.001)
Flashbacks	1425	28.1	1023	20.2	402	28.2	20.05 (<.001)
Internal avoidance	1891	37.3	1573	31.0	318	16.8	17.83 (<.001)
External avoidance	1714	33.8	1444	28.5	270	15.8	16.43 (<.001)
Hypervigilance	2114	41.7	1247	24.6	867	41.0	29.45 (<.001)
Hyperarousal	1833	36.2	1235	24.4	598	32.6	24.45 (<.001)
Difficulty calming down	2096	41.3	1450	28.6	646	30.8	25.42 (<.001)
Emotionally numb	1554	30.7	923	18.2	631	40.6	25.12 (<.001)
Feel like a failure	1489	29.4	1113	22.0	376	25.3	19.39 (<.001)
Feel worthless	1381	27.2	1069	21.1	312	22.6	17.66 (<.001)
Feel cut off from others	1485	29.3	1189	23.5	296	19.9	17.21 (<.001)
Difficult close to others	1618	31.9	1266	25.0	352	21.8	18.76 (<.001)
***Symptom clusters***							
Re-experiencing	1815	35.8	1214	23.9	601	33.1	24.52 (<.001)
Avoidance	2236	44.1	1945	38.4	291	13.0	17.06 (<.001)
Sense of current threat	2509	49.5	1672	33.0	837	33.4	28.93 (<.001)
Functional impairment (PTSD symptoms)	1936	38.2	1283	25.3	653	33.7	25.55 (<.001)
Affective dysregulation	2468	48.7	1758	34.7	710	28.8	26.65 (<.001)
Negative self-concept	1725	34.0	1347	26.6	378	21.9	19.44 (<.001)
Disturbed relationships	1960	38.7	1609	31.7	351	17.9	18.74 (<.001)
Functional impairment (DSO symptoms)	1880	37.1	1282	25.3	598	31.8	24.45 (<.001)
***Disorders***							
PTSD	365	7.2	195	3.8	170	46.6	10.09 (<.001)
CPTSD	606	12.0	300	5.9	306	50.5	17.49 (<.001)
PTSD or CPTSD	971	19.2	495	9.8	476	49.0	21.82 (<.001)

The clinical checks reduced endorsements for all PTSD and DSO symptoms (all *p*s < .001), with relative reductions ranging from 15.8% (external avoidance) to 51.6% (nightmares). Endorsement of all six symptom clusters also decreased significantly (all *p*s < .001), with relative reductions ranging from 13.0% (avoidance) to 33.4% (sense of current threat).

At the diagnostic level, 19.2% of participants met requirements for *ICD-11* PTSD or CPTSD without the clinical checks, falling to 9.8% with them (relative reduction = 49.0%, *Z* = 21.82, *p* < .001). Requirements for PTSD were met by 7.2% without the clinical checks and 3.9% with them (relative reduction = 45.8%, *Z* = 10.09, *p* < .001), while requirements for CPTSD were met by 12.0% without them and 5.9% with them (relative reduction = 50.5%, *Z* = 17.49, *p* < .001).

### Objective 3 results

When the threshold for symptom and functional impairment endorsement on the standard ITQ was raised from 2 to 3 on the Likert scale, 4.8% of participants screened positive for either disorder (2.4% for PTSD and 2.4% for CPTSD). These proportions were significantly lower than those obtained using the ITQ-CC scoring rules for either disorder (9.8% vs. 4.8%, *Z* = 13.32, *p* < .001), PTSD (3.8% vs. 2.4%, *Z* = 5.02, *p* < .001), and CPTSD (5.9% vs. 2.4%, *Z* = 11.89, *p* < .001). Agreement between the two approaches to identifying diagnostic cases was in the moderate-to-substantial range: PTSD (κ = .47), CPTSD (κ = .63), and either disorder (κ = .63).

### Objective 4 results

Table 7 presents the associations between *ICD-11* PTSD and CPTSD diagnostic status, estimated with and without the clinical checks, and the three criterion variables (lifetime history of suicidal ideation, attempted suicide, and ‘severe’ anxiety-depression symptoms). In every case, associations were stronger when diagnostic status was based on the clinical checks. Bootstrapped tests of the paired difference confirmed that all six increases in association were statistically significant (*p*s < .05). Notably, the association between PTSD and anxiety-depression was significant only when estimated using the clinical checks.

**Table 7:** Associations between ICD-11 PTSD and CPTSD diagnostic status estimated with and without the clinical checks (CC) and three clinical criterion variables.

	Anxiety-depression ^1^	Suicidal ideation ^2^	Suicide attempt ^2^
	OR (95% CI)	OR (95% CI)	OR (95% CI)
**ICD-11 PTSD**			
Without CC	1.28 (0.93, 1.75)	1.37^**^ (1.08, 1.74)	1.46^*^ (1.06, 2.01)
With CC	2.47^***^ (1.75, 3.50)	2.14^***^ (1.55, 2.93)	2.46^***^ (1.68, 3.60)
**ICD-11 CPTSD**			
Without CC	7.33^***^ (6.00, 8.94)	4.76^***^ (3.89, 5.81)	4.06^***^ (3.23, 5.10)
With CC	8.97^***^ (7.00, 11.49)	6.06^***^ (4.54, 8.07)	5.26^***^ (3.96, 7.00)

*Note.* CC = clinical checks; OR = odds ratio; CI = confidence interval. Anxiety-depression was defined as a score in the severe range (9–12) on the PHQ-4. ^1^ = Sample size = 5070, ^2^ = sample size = 4053. The suicidality items were not administered in the Czech Republic. * p < .05. ** p < .01. *** p < .001.

Because the clinical checks strengthened the association between PTSD and each criterion variable, unplanned post-hoc analyses were conducted to examine whether the expected stronger associations for CPTSD relative to PTSD with all criterion variables was diminished with the clinical checks. All three criterion variables remained associated with a greater likelihood of meeting requirements for CPTSD rather than PTSD, both without and with the clinical checks: anxiety-depression (OR = 3.81 vs 2.96), suicidal ideation (OR = 2.88 vs 2.60), and suicide attempt (OR = 2.23 vs 1.90). The differential associations narrowed but remained, consistent with the clinical checks improving the identification of PTSD cases rather than eroding the distinction between the two disorders.

### Objective 5 results

Of the 971 participants who met diagnostic requirements for *ICD-11* PTSD or CPTSD on the standard ITQ, 476 (49.0%) lost their probable diagnostic status when the clinical checks were used, while 495 (51.0%) retained their probable diagnostic status. The binary logistic regression model identifying factors associated with losing versus retaining probable diagnostic status was therefore based on 971 participants and was statistically significant (*χ*^*2*^ (14) = 120.62, *p* < .001), explaining between 11.7% (Cox and Snell R^2^) and 15.6% (Nagelkerke R^2^) of ‘variance’ in the criterion variable. Results are presented in Table 8.

**Table 8:** Binary logistic regression predicting loss of probable diagnostic status when the clinical checks were applied (n = 971).

	Wald	OR	95% CI	*p*
Female (vs. male)	7.11	0.68	0.51, 0.90	.008
Age	0.01	1.00	0.99, 1.01	.924
In a relationship	0.76	0.88	0.66, 1.18	.385
University education	3.30	0.75	0.55, 1.02	.069
Unemployed	0.35	0.88	0.56, 1.36	.553
Number of children	0.00	1.00	0.87, 1.15	.986
Annual income	0.10	0.99	0.92, 1.07	.753
Time since index trauma	1.61	1.08	0.96, 1.21	.205
Number of trauma types	22.04	0.94	0.91, 0.96	<.001
Anxiety-depression	53.86	0.84	0.80, 0.88	<.001
Hungary	0.37	1.17	0.71, 1.91	.543
Poland	2.14	1.36	0.90, 2.04	.144
Romania	0.32	0.87	0.54, 1.41	.574
Slovakia	0.93	1.25	0.80, 1.95	.334

*Note*. OR = odds ratio; CI = confidence interval. The criterion variable was coded 0 = retained probable diagnostic status (n = 495), 1 = lost probable diagnostic status (n = 476).

Three variables were significantly associated with losing probable diagnostic status. Females were less likely than males to lose diagnostic status (OR = 0.68, *p* = .008). Each additional type of traumatic event reported reduced the odds of losing diagnostic status by approximately 6% (OR = 0.94, *p* < .001), and each one-point increase in anxiety-depression symptoms on the PHQ-4 reduced the odds of losing diagnostic status by approximately 16% (OR = 0.84, *p* < .001). Country was not associated with losing diagnostic status, indicating that the clinical checks functioned comparably across the five national samples.

## Discussion

This study was carried out to systematically evaluate the performance of the recently developed clinical checks for the ITQ across five objectives. Overall, these results indicate that the ITQ with clinical check items retain the same excellent psychometric properties of the standard ITQ; capture clinically meaningful information beyond that which is obtained by simply raising the endorsement threshold on the standard ITQ; yield diagnostic classifications more strongly associated with clinically relevant external criteria; generate substantially lower, and arguably more plausible, prevalence estimates for *ICD-11* PTSD and CPTSD in the general adult population; and retain those individuals with a greater trauma burden and concurrent psychological distress as diagnostic cases.

Before discussing the individual results, it is important to address what is likely to be the most common critique of these findings. Some readers may object that in the absence of clinical interview data, there was no way to judge the accuracy of the prevalence estimates derived from the ITQ with clinical checks. However, this critique rests on the assumptions that the clinical interview method yields accurate estimates of disorder prevalence, and more accurate estimates of prevalence than self-report assessments, and there is no evidence to support either of these assumptions [26]. There is evidence that clinical interviews often yield *different* prevalence estimates to self-report questionnaires [20–25], but different does not mean more accurate. Since there is no objective test for the presence of PTSD, CPTSD, or any other psychiatric disorder, it is not possible to prove that a prevalence estimate derived from a clinical interview more closely approximates the true prevalence rate of a disorder than an estimate derived from a self-report questionnaire. Clinical interviews are moreover subject to their own measurement errors and biases, and often much greater than those associated with a self-report questionnaire [26]. Consequently, even if clinical interviews had been conducted with all participants, we still would not have been able to say anything definitive about the accuracy of the rates reported using the clinical checks.

The first objective of the current study was to examine the psychometric properties of the scores obtained using the ITQ with clinical checks. The CFA results indicated that the latent structure of these data was consistent with the *ICD-11* formulation of PTSD and CPTSD (i.e., the distinction between the PTSD and DSO symptom sets). The correlated six-factor model accurately represented the sample data, and all six subscales evidenced satisfactory internal reliability. Notably, the two-factor second-order model contained one out-of-bounds parameter estimate (i.e., affective dysregulation loading onto DSO above 1.0); a finding that has been reported previously in some studies with the standard ITQ [34–36]. This problem was addressed by allowing the affective dysregulation factor to cross-load onto the second-order PTSD factor. This has been explained by the fact that the causal processes involved in the development of the PTSD symptoms also contribute to the development of the hyperactivation component of the affective dysregulation cluster [43,44]. On this account, the hyperactivation symptom in the affective dysregulation cluster serves as a kind of bridge symptom between the PTSD and DSO symptoms in CPTSD. This was also observable in the LCA results. While qualitatively distinct groups of participants with symptom profiles reflective of *ICD-11* PTSD and CPTSD were observed, readers will note that the PTSD class was characterised by a somewhat elevated probability of endorsing the Affective Dysregulation symptom cluster. Overall, these results indicate that adding the clinical checks does not compromise the psychometric properties of the ITQ.

Regarding the study’s second objective, the clinical checks produced statistically significant reductions in endorsement rates for all symptoms and symptom clusters. Averaged across all symptom items, the relative reduction was 31% for the six PTSD symptoms and 27% for the six DSO symptoms. At the cluster level, reductions were somewhat larger for the three PTSD clusters (26.5%) than for the three DSO clusters (22.9%). This translated into a reduction in overall diagnostic cases of 49.0%, with similar reductions observed for PTSD (46%) and CPTSD (51%). In their study of UK adults, Shevlin et al. [18] reported reductions of 42% overall, and 30% and 48% for PTSD and CPTSD, respectively. Taken together, these findings indicate that in quota-matched samples drawn from the general adult population, the clinical checks reduce overall prevalence rates by about 40–50%, with substantial effects on the estimated prevalence rates of PTSD and CPTSD.

It was striking that nearly 20% of participants met all diagnostic requirements for *ICD-11* PTSD or CPTSD based on the standard ITQ scoring rules. It is quite unrealistic that one-in-five adults living in these European nations is suffering from a posttraumatic stress disorder. Part of this is almost certainly attributable to sampling: as is made clear in Table 1, quota-matched samples of the kind used in the current study consistently yield higher estimates than probability-based samples. When the clinical checks were applied, the overall rate fell to 9.8%, with 3.8% meeting requirements for PTSD and 5.9% for CPTSD; figures that sit close to the averages from across all studies listed in Table 1. This suggests that the clinical checks may well be identifying and removing false-positive diagnostic cases. This should obviously be interpreted cautiously since the estimates in Table 1 are themselves derived from self-report questionnaires without clinical checks, so agreement with them cannot establish that the present estimates are accurate. Nevertheless, what these results do indicate is that the clinical checks brought implausibly high estimates of prevalence into the range that is expected based on the broader empirical literature.

The third and fourth objectives of this study were to test if the clinical checks were doing what they were designed to do (i.e., adding clinically relevant information to the measurement of PTSD and DSO symptoms). We tested this in two ways. First, we compared the proportion of people who screened positive for PTSD and/or CPTSD with the clinical checks to the proportion who screened positive when the threshold for symptom endorsement on the standard ITQ was set one point higher on the Likert scale. If the clinical checks functioned simply as an indirect way of raising the intensity threshold for symptom endorsement, then the two approaches should have identified similar proportions and, more importantly, the same individuals. Neither was the case. It can therefore be concluded that the clinical checks are not simply increasing the severity threshold required for symptom endorsement.

The second way we attempted to test if the clinical checks were adding clinically relevant information was to examine how diagnostic status with and without the clinical checks related to three clinically meaningful external variables. Without the clinical checks, PTSD and CPTSD correlated positively and significantly with variables representing clinically significant levels of anxiety-depression symptoms, lifetime suicidal ideation, and lifetime attempted suicide (the only exception being the association between PTSD and anxiety-depression symptoms), signifying that the standard ITQ estimates are indeed clinically meaningful. However, using the clinical checks, these correlations were all markedly stronger, and the association between PTSD and anxiety-depression symptoms became significant. These results are consistent with the view that the clinical checks are adding clinically meaningful information to the standard ITQ symptom assessments. Moreover, consistent with the understanding of CPTSD as the more burdensome disorder, all three criterion variables were more strongly associated with CPTSD than PTSD, both with and without the clinical checks. As such, the expected differences in risk for PTSD versus CPTSD was maintained when the clinical checks were used.

The final objective was to explore why some people lose their probable diagnostic status when the clinical checks were used. We identified three variables that were independently associated with losing diagnostic status: sex, the number of different traumatic life events experienced, and anxiety-depression symptoms. Men were more likely than women to lose their diagnostic status; those who experienced fewer different traumatic life events were more likely to lose their diagnostic status; and those with lower levels of anxiety-depression symptoms were more likely to lose their diagnostic status. What these latter two results reveal is that that the people who retained their diagnosis were those with more evidence of traumatization and concurrent psychological distress; a finding consistent with the proposition that the clinical checks are identifying and removing false-positive diagnostic cases. The finding that men were more likely to lose their diagnostic status is less easy to explain, but one possibility is that the men, due to higher average levels of trait impulsivity compared to women [45,46], may be more likely to initially report a symptom and then correct their initial report when probed for further clarifying information. Notably, the absence of any effect for nationality in these analyses suggests that the clinical checks are operating similarly irrespective of nationality or language.

Current findings should be interpreted considering several limitations. First, the cross-sectional nature of the data means that no causal conclusions can be drawn about the nature of the reported relationships between the study variables. Second, the quota-matched sampling method means that the national samples are unlikely to be entirely representative of the populations from which they were drawn, meaning that generalisations are necessarily limited. Third, we only examined a small number of variables that might explain why people lost their probable diagnostic status with the clinical checks. More work will be required to better understand this phenomenon. Fourth, participants were classified as having ‘clinically relevant’ levels of anxiety-depression based on their PHQ-4 scores. While this is an excellent measure, we recognize that it is not a substitute for a comprehensive assessment of either generalized anxiety disorder or major depressive disorder, meaning these results should be interpreted cautiously.

## Conclusions

These findings provide new and pertinent information about the nature and value of using clinical checks within the ITQ to measure PTSD and DSO symptoms. Supplementing the standard ITQ with clinical checks produced scores with excellent psychometric properties, that captured additional, clinically relevant information beyond the standard ITQ assessment items, and yielded more plausible estimates of disorder prevalence, seemingly by retaining as diagnostic cases those with more extensive trauma histories and concurrent mental distress. Results were, therefore, consistent with the assumption that the clinical checks are removing probable false-positive diagnostic cases. The use of clinical checks in self-report questionnaires like the ITQ represents a new and potentially valuable way of assessing for the presence of psychiatric disorders. We hope this work motivates others to continue exploring the value of the clinical checks within the ITQ, but also to explore the usefulness of this approach in the assessment of other psychiatric disorders.
